# Identifying Determinants of Socioeconomic Inequality in Health Service Utilization among Patients with Chronic Non-Communicable Diseases in China

**DOI:** 10.1371/journal.pone.0100231

**Published:** 2014-06-24

**Authors:** Xin Xie, Qunhong Wu, Yanhua Hao, Hui Yin, Wenqi Fu, Ning Ning, Ling Xu, Chaojie Liu, Ye Li, Zheng Kang, Changzhi He, Guoxiang Liu

**Affiliations:** 1 Department of Social Medicine, School of Health Management, Harbin Medical University, Harbin, Heilongjiang, People's Republic of China; 2 Department of Humanities and Social Sciences, Harbin Medical University (Daqing), Daqing, Heilongjiang, People's Republic of China; 3 Centre of Health Statistics and Information, Ministry of Health, Beijing, People's Republic of China; 4 Modern Health Management Technique Development and Application Laboratory, Harbin Medical University, Harbin, Heilongjiang, People's Republic of China; 5 School of Public Health, La Trobe University, Melbourne Victoria, Australia; Iran University of Medical Sciences, Islamic Republic Of Iran

## Abstract

**Background:**

People with chronic non-communicable diseases (NCD) are particularly vulnerable to socioeconomic inequality due to their long-term expensive health needs. This study aimed to assess socioeconomic-related inequality in health service utilization among NCD patients in China and to analyze factors associated with this disparity.

**Methods:**

Data were taken from the 2008 Chinese National Health Survey, in which a multiple stage stratified random sampling method was employed to survey 56,456 households. We analyzed the distribution of actual use, need-expected use, and need-standardized usage of outpatient services (over a two-week period) and inpatient services (over one-year) across different income groups in 27,233 adult respondents who reported as having a NCD. We used a concentration index to measure inequality in the distribution of health services, which was expressed as HI (Horizontal Inequity Index) for need-standardized use of services. A non-linear probit regression model was employed to detect inequality across socio-economic groups.

**Results:**

Pro-rich inequity in health services among NCD patients was more substantial than the average population. A higher degree of pro-rich inequity (HI = 0.253) was found in inpatient services compared to outpatient services (HI = 0.089). Despite a greater need for health services amongst those of lower socio-economic status, their actual use is much less than their more affluent counterparts. Health service underuse by the poor and overuse by the affluent are evident. Household income disparity was the greatest inequality factor in NCD service use for both outpatients (71.3%) and inpatients (108%), more so than health insurance policies. Some medical insurance schemes, such as the MIUE, actually made a pro-rich contribution to health service inequality (16.1% for outpatient and 12.1% for inpatient).

**Conclusions:**

Inequality in health services amongst NCD patients in China remains largely determined by patient financial capability. The current insurance schemes are insufficient to address this inequity. A comprehensive social policy that encompasses a more progressive taxation package and redistribution of social capital as well as pro-poor welfare is needed.

## Background

In recent decades, China has achieved unprecedented success in economic development largely due to market reforms. Unfortunately, the rapid growth of available wealth has not been distributed evenly across the population, resulting in a widening wealth gap between rich and poor and increased disparity in health service utilization. Although people had started to recognize health equality disparities [1], many were astonished when the 2000 Health System Performance Report published by the World Health Organization (WHO) ranked China as one of the lowest three among 191 countries in terms of fairness of health financing. Indeed, in 2001 Chinese people paid 60% of total health expenditure (THE) out of pocket (OOP) [2]. This exacerbated the pre-existing financial obstacles regarding access to health care services, forcing more people, especially those living in poverty [3], to forego much needed medical services. The 2008 National Health Services Survey (NHSS) revealed that among constituents of non-treatment: 10.6% of people did not seek medical attention they needed over a two–week period, 25.1% of people refused inpatient services recommended or prescribed by doctors. Of these two groups, 29.2% and 70.3% refused or avoided treatment due to financial difficulties [4]. Equity of access to healthcare in the Chinese healthcare system has since become a serious concern [5]–[7].

People with chronic non-communicable diseases (NCD) are particularly vulnerable to health inequities due to long-term and often expensive health care needs, compounded by an inability to earn more money. A 2003 study estimated that in China NCD associated costs amounted to $123,548 Billion (USD), which accounted for 71.45% of the financial burden of all diseases and 7.31% of GDP [8]. The burden of NCD was expected to continue to rise [9]. By 2010, the number of patients with diagnosed hypertension in China exceeded 200 million [10], while those with diabetes mellitus numbered 92 million [11]. It was estimated that cardiovascular diseases alone accounts for 41% of all deaths in China [12]. In total, NCD contributed to 80% of deaths and 70% of disability-adjusted life-years (DALYs) lost in China [9]. Previous studies showed that people living with NCD were more susceptible to medical impoverishment [13]. International evidence suggests that people of lower economic status often demonstrate lower rates of access to medical services when compared with those more affluent [14]–[16], especially among those with NCD [17]–[19]. People with NCD often lack access to or eligibility for affordable medical insurance [20], [21]. Consequently, those who do seek medical services have to pay large co-payments, which they can often ill afford, and so compound their socioeconomic status - keeping them in poverty [19], [22], [23].

Previous studies in China have failed to determine the full extent of nation-wide inequality of access to health services [18], [24]. With limited aggregated data, most studies have focused on either regional disparity in relation to a specific service [7], [25]–[29] or income differences in service utilization within a given general population (urban [6], [30]–[33] or rural [18], [34], [35]). We believe that it is likely that inequality of access may have been underestimated by failing to take into account study subjects with and without NCD. People with NCD often require more frequent visits to health facilities in the long term, and are more likely to eschew health services if financial difficulties arise. Unfortunately, there is a paucity of literature documenting inequality in access to health services among NCD patients in China. This study aims to meet this need by determining the degrees of inequality in health services access amongst people with NCD in China, and identifying factors that may be associated with this inequality.

## Methods

### Data source

The data collected from the 2008 National Health Services Survey (NHSS) were used for this study. The NHSS is one of the most representative health surveys in China organized by the Ministry of Health. It is conducted every five years (in 1993, 1998, 2003 and 2008). The surveys used a questionnaire collecting data in relation to demographic characteristics, income, health status, medical service utilization and medical expenses of those surveyed.

A total of 177,501 questionnaires were completed in the 2008 NHSS, involving 56,456 households across 31 provinces in China. For this study, respondents of 15 years old and over (143,214) were eligible: of these, 27,233, or 19.01% reported having had NCD.

### Sampling method

A four-stage stratified random sampling strategy was employed to maximize the representation of the social and economic characteristics of the entire population of China. In the first stage, 94 counties/cities were proportionally and randomly selected, representing five social economic zones. In the second stage, five rural townships in each county and five urban districts in each city were chosen at random. In the third stage, two villages in each rural township and two neighborhoods in each urban district were randomly selected. In the fourth stage, 60 households were randomly selected in each village or neighborhood.

A questionnaire survey was administered via face-to-face interviews with household members. Written informed consent was obtained prior to the survey. For those unable to give informed consent, for example those under 15 years or those with intellectual disability, consent were sought from their next of kin, carers, or parents. The database used in this study contained de-identified data to protect the privacy of participants.

### Ethics statement

Ethics clearance was obtained from the Medical Ethics Committee of Harbin Medical University (Da Qing). The authors declare no conflict of interest.

### Measurements

#### Dependent variables

NCD-associated use of services was measured by two binary variables: (1) use (yes or no) of outpatient care over a two week period and (2) use (yes or no) of inpatient care over a 12 month period. Respondents were asked: “Have you received any medical treatment during the last two weeks?” and “Have you been admitted to hospital during the past year?” Those who answered “yes” to the above questions were asked to explain reasons for the treatment or/and hospitalization (including duration of conditions and diagnosis in relation to those conditions). The reported reasons were coded and classified into NCD-associated and non-NCD-associated conditions.

NCD was defined as a chronic medical condition diagnosed by a doctor at least six months before the survey, for which either the symptom(s) persisted or relevant medical treatment continued. In this study, the chronic conditions identified included hypertension, cardiovascular disease, cancer, chronic obstructive pulmonary disease, diabetes mellitus and cerebrovascular disease, which accounted for 88% of all reported NCDs.

#### Independent and control variables

Health service usage is associated not only with response to disease, age, sex, self-assessed health and limitation of daily activities (need variables), but also with other factors such as socioeconomic status (non-need variables). In this study, we considered household income, education, marital status, occupation, health insurance policies, distance to health facilities, and location of residence as non-need variables.

Age was categorized into five year groups until 65 years. Self-assessed health was measured using a single question on a five-point Likert scale. Limitation of daily activities was measured as “yes” or “no” in relation to the experience of survey participants over the past 12 month prior to the survey. Household income was measured using per capita consumption expenditure and was equally divided into five groups, ranging from the poorest to the richest. Education attainment was measured as the maximum education level attained by the survey participants. Occupation was categorized using the Chinese government standards (student, farmer, worker, self-employed, manager/professional/clerk, unemployed and others). Five major health insurance policies were identified: Free Medical Care (FMC) for governmental officials and senior veterans, Medical Insurance for Urban Employees (MIUE), Medical Insurance for Urban Residents (MIUR), New Cooperative Medical Insurance Scheme (NCMS), and others. The NCMS is an insurance scheme designed specifically for rural residents. By 2008, more than 90% of rural residents had been covered by the NCMS[36]. Participant residence was classified as either eastern (wealthier), middle, or western (poorer) regions. All independent and control variables were coded as dummy variables for further analysis.

### Statistical analysis

The World Health Organization defines health equity as “the absence of avoidable or remediable differences among groups of people” [37]. Equality is an aspirational ideal, an equal distribution of *access* to health services across groups of people. When differential need has been adjusted, an inequality can be interpreted as inequity [38]. A common interpretation of equity in health utilization is that health care ought to be allocated according to health need rather than characteristics of non-need variables, such as income or residence [39]. The conceptual literature on equity in health utilization distinguishes between horizontal and vertical equity. This study is focused on horizontal equity, that is, persons with equivalent need for healthcare ought to have access to treatment irrespective of their socioeconomic status [39].

We calculated distributions of actual use of services, need-expected use of services and need-standardized use of services across income groups. The actual use of services is a factual depiction of the extent of equality (or inequality) in the distribution of services; whereas, the need-expected use of services is a predicted use of services by the "need variables". The gap between “actual use of services” (a) and “need-expected use of services” (b) reflects underuse (if a<b) or overuse (if a>b) of services. However, the combination of the above two measurements does not quantify the extent of inequity. Inequity can be reflected from need-standardized use of services. The aim of the standardization is to determine how the actual distribution of use of services would appear in the absence of differences in the distribution of health need [40]. An unequal distribution in need-standardized use of services can be explained as horizontal inequity because it implies that persons with the same needs are not treated the same.

This study used an indirect standardization method to calculate the distribution of need-standardized use of services. It required estimates of the distribution of the standardizing variables (x) and the variable of interest (y), and the correlation between these variables [40]. A non-linear probit regression model was employed to estimate the need-standardized use of service.

The non-linear regression proceeded by an equation for estimating the actual use of services (y_i_):
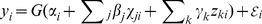
(1)then a linear approximation of this function was given by:
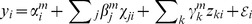
(2)where *i* denotes the individual; χ*_j_* are need variables for which we want to standardize; and the 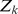 are non-need variables for which we do not want to standardize but to control in the estimation of the 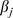, 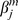 are the marginal effects of a set of j need. The 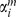 is an intercept; 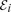 is the implied error term which includes approximation errors [38]; and 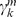 are the marginal effects of a set of k non-need variables.

Estimates of the coefficients in (1) were then combined with actual values of the x variables and sample means of the z variables [38] to obtain the distribution of need-expected use of services (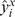).
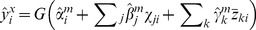
(3)


Estimates of indirectly need-standardized use of services (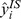) were obtained as the difference between actual and x-expected use, plus the mean of predictions (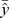).
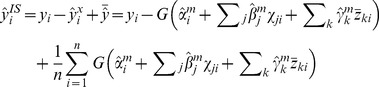
(4)


Where 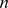 is the sample size, and we have chosen to set 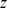 variables (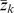) to their means, in obtaining the predictions.

The methods used in this study were identical to those proposed by Wagstaff et al [41]. We used a concentration curve to depict inequality. A concentration curve plots the cumulative percentage of use of services (y-axis) against the cumulative percentage of respondents, ranked by household income, beginning with the least affluent and ending with the most affluent (x-axis). If every person, irrespective of his or her income, has exactly the same use of services, the concentration curve will be a 45 degree line, running from the bottom left-hand corner to the top right-hand corner. This is known as the line of equality. The farther the concentration curve lies from the line of equality, the greater the degree of inequality [42], [43].

We calculated concentration index (CI) to measure socioeconomic inequality in use of services according to Equation (5) [44]. 
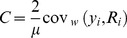
(5)where *y_i_* is the measure of actual use of services and *R_i_* is the relative fractional rank (based on weights) of the i^th^ individual which indicates the weighted cumulative proportion of the population up to the midpoint of each individual weight. The* µ* is the (weighted) mean of use of services and *cov_w_* denotes the weighted covariance.

The CI was defined as twice the area between the concentration curve and the line of equality, which quantitatively reflects the degree of equality. It lies in the range of -1 to +1, with a negative (positive) value representing inequality in favor of groups with lower (higher) income. Zero indicates that there is no inequality. The greater the absolute value of concentration index, the worse off for the disadvantaged groups of people.

The method proposed by Wagstaff et al. [43] was employed to decompose socioeconomic inequality in use of services into individual determinants. A decomposition analysis estimates how determinants proportionally contribute to inequality in the use of services [44].

Because “use of services” was measured as a binary variable in this study, we applied a nonlinear probit regression model. By using a nonlinear model; however, decomposition can only be made via some linear approximation (a linear approximation to equation (1) is expressed as equation (2). The overall inequalities in use of services (C) is expressed as [43].
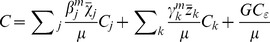
where 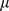 is the mean of 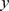; 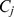 and 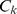 are the concentration index of 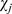 and 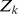; and 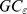 is the generalized concentration index of the error tem 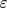 [43].

The overall inequality in an outcome contains two components: an explained component and an unexplained component. In the explained component, the impact of each determinant on an outcome is measured by its elasticity (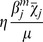 or 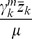) and the extent of unequal distribution of each determinant across income groups is measured by (CI) [21]. The absolute contribution of each determinant is calculated by multiplying the η and CI with respect to that determinant. An unexplained component is residual which reflects the inequality in health that can't be explained by systematic variation.

All analyses were performed with the STATA software version 10.

## Results

### 1. Social demographic characteristics of respondents

Our study subjects (respondents with NCD) had differing social demographic profiles compared with the total adult respondents. On average they were more likely to be older, in poorer health, have a lower level of education, unemployed, divorced or widowed compared to the total adult respondent population (Table 1). However, respondents reporting NCD were slightly more likely to be covered by health insurance than the average of the adult respondents (90.8% vs 88.4%).

**Table 1:** Social demographic characteristics and healthcare accessibility of respondents.

Variables	All adult respondents (n = 143,214)		Subjects of study (n = 27,233)	
	Number	(%)	Number	(%)
**Gender and Age (years)**				
** Men** *				
15–24	11,026	7.7	236	0.9
25–34	9,744	6.8	401	1.5
35–44	15,223	10.6	1,404	5.2
45–54	14,010	9.8	2,618	9.6
55–64	10,795	7.6	3,276	12.00
65-	9,693	6.8	4,316	15.9
** Women** *				
15–24	10,764	7.5	194	0.7
25–34	10,449	7.3	550	2.0
35–44	16,182	11.3	1,930	7.1
45–54	14,324	10.0	3,460	12.7
55–64	10,679	7.5	3,804	14.0
65-	10,325	7.1	5,044	18.4
**Health Status**				
** Limitation of daily Activities** *				
Yes	7,808	5.4	5,095	18.7
No	135,406	94.6	22,138	81.3
** Self-rated health** *				
Very poor	478	0.3	364	1.3
Poor	2,730	1.9	1,847	6.8
Fair	16,150	11.3	8,024	29.5
Good	59,950	41.9	13,533	49.7
Excellent	63,906	44.6	3,465	12.7
**Socioeconomic Status**				
** Education** *				
Illiterate	22,107	15.4	7,222	26.5
Primary school	88,800	62.0	15,556	57.1
Secondary school	22,927	16.0	3,195	11.8
University	9,380	6.6	1,260	4.6
** Occupation** *				
Student	9,854	6.9	120	0.4
Unemployed	21,773	15.2	6,486	23.8
Peasant	76,224	53.2	12,246	45.0
Worker	6,079	4.2	1,904	7.0
Self-employed	8,529	6.0	1,224	4.5
Manager/Professional/Clerk	18,139	12.7	4,780	17.6
Other	2,616	1.8	473	1.7
** Marital status** *				
Unmarried	23,243	16.2	983	3.6
Married	107,462	75.0	21,487	78.9
Divorced/widowed	12,159	8.5	4,713	17.3
Other	350	0.3	50	0.2
** Household income**				
Quintile I (poorest)	28,649	20.0	5,453	20.0
Quintile II	28,957	20.2	5,451	20.0
Quintile III	28,325	19.8	5,473	20.1
Quintile IV	28,643	20.0	5,417	19.9
Quintile V (richest)	28,640	20.0	5,439	20.0
** Location of residence** *				
East	49,466	34.5	10,269	37.7
Middle	40,424	28.2	7,718	28.3
Western	53,324	37.3	9,246	34.0
**Healthcare Accessibility**				
**Insurance scheme** *				
MIUE	22,106	15.4	6,268	23.0
FMC	1,674	1.2	607	2.2
MIUR	5,021	3.5	846	3.1
NCMS	96,634	67.5	16,822	61.8
Other insurance	1,137	0.8	178	0.7
No insurance	16,642	11.6	2,512	9.2
**Distance to the nearest health facilities** *				
≤5 km	136,146	95.1	26,056	95.7
>5 km	7,068	4.9	1,177	4.3
**Time to the nearest health facilities** *				
≤30 min	137,071	95.7	26,161	96.1
>30 min	6,143	4.3	1,072	3.9
**HI of outpatient service (HI_o_)**	0.017		0.089	
**HI of inpatient service (HI_i_)**	0.207		0.253	
**Average Medical Expense Per Outpatient(US$)**	24.3		70.4	
**Average Medical Expense Per Inpatient(US$)**	728.3		1056.7	

*p<0.05, compared between the study population (people with NCD) and the average of adult respondents.

MIUE, Medical Insurance for Urban Employees;

FMC, Free Medical Care.

MIUR, Medical Insurance for Urban Residents.

NCMS, New Cooperative Medical Insurance Scheme for Rural Residents.

Respondents with NCD reported more expensive medical costs: their outpatient and inpatient expenses were respectively 2.9 and 1.5 times higher than average. The concentration index for need-standardized use of services (expressed as HI) proved our assumption that people with NCD had a higher degree of inequity in both outpatient services (0.089 vs 0.017) and inpatient services (0.253 vs 0.207) as compared with the average of adult respondents.

### 2. Distribution of health services

**Figure 1:**
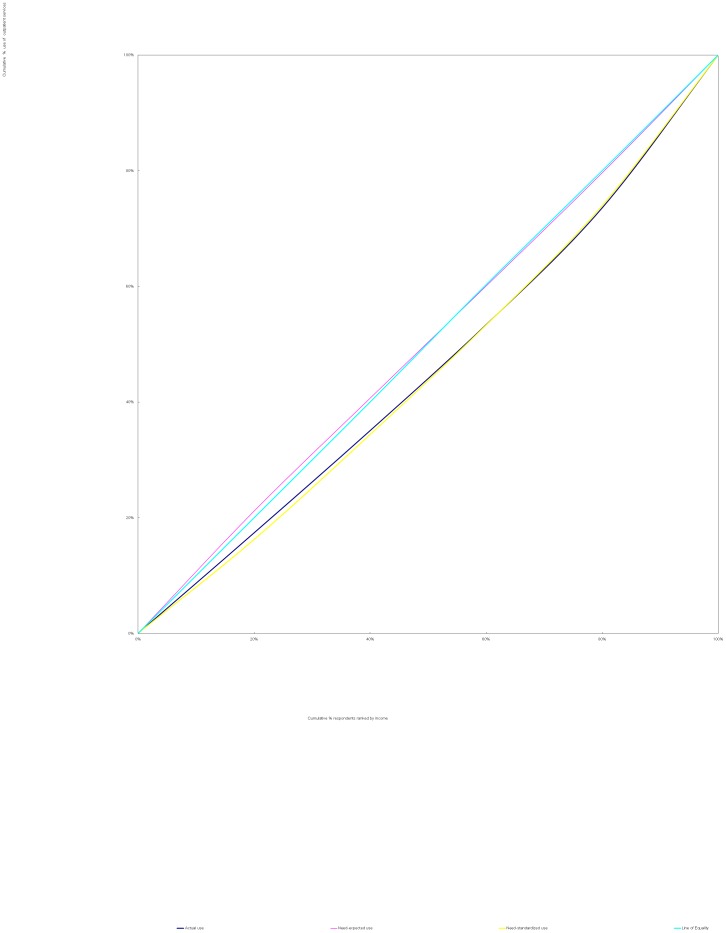
Concentration curves for use of outpatient services, China 2008. The line of need-expected services lie nearer to equality line. Both lines of actual use and of need-standardized services lie below the equality line and almost coincide with each other.

**Figure 2:**
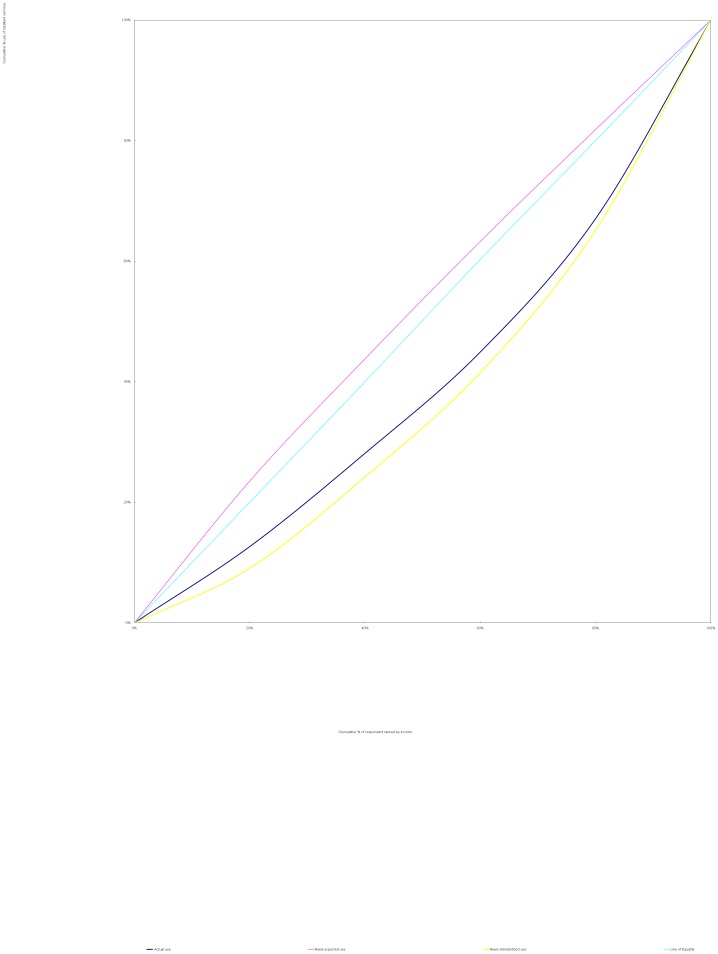
Concentration curves for use of inpatient services, China 2008. The line of need-expected services lie above the equality line. Both lines of actual use and of need-standardized services lie below the equality line. The line of need-standardized services lie farther away from the equality line than that of actual use.

Significant income-related inequality as measured by CI was found in actual, need-expected and need-standardized use of outpatient and inpatient services (*p*<0.05). There was a pro-poor inequality in need-expected use of services(From Figure 1 and Figure 2, greater need of services from the poor was demonstrated by the lines of need-expected services, which lie above the equality lines), indicating that NCD patients with lower income had greater needs for both outpatient (C_N_ = −0.006) and inpatient (C_N_ = −0.052) services. However, pro-rich inequality in actual and need-standardized use of services (From Figure 1 and Figure 2, both lines of actual use and of need-standardized services lie below the equality lines) was found. Greater CI (inequality) was found for use of inpatient services (C_M_ = 0.201 for actual use; HI = 0.253 for need-standardized use) as compared with outpatient services (C_M_ = 0.083 for actual use; HI = 0.089 for need-standardized use) (Table 2). This indicates that the NCD patients with lower income used less services despite a greater need compared with their richer counterparts, and such inequality was more substantial in inpatient services than in outpatient services. As seen from Figure 1 and Figure 2, the lines of actual use and of need-standardized services for inpatient services lie farther away from the equality lines than those for outpatient services.

**Table 2:** Distribution of actual, need-expected and need-standardized NCD use of outpatient and inpatient services across household income quintiles.

Household income	Outpatient service use			Inpatient service use		
	Actual ( 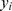 )	Need–Expected ( 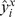 )	Need-Standardized ( 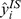 )	Actual ( 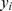 )	Need–Expected( 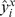 )	Need-Standardized ( 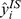 )
**Quintile I (Poorest)**	0.089	0.109	0.084	0.043	0.081	0.031
**Quintile II**	0.091	0.100	0.093	0.053	0.070	0.052
**Quintile III**	0.094	0.100	0.097	0.058	0.067	0.059
**Quintile IV**	0.104	0.101	0.106	0.076	0.064	0.081
**Quintile V (Richest)**	0.136	0.105	0.134	0.113	0.063	0.119
**C_M_/C_N_/HI**	**0.083**	**−0.006**	**0.089**	**0.201**	**−0.052**	**0.253**
**SrErr**	0.011	0.002	0.011	0.014	0.003	0.013
**T**	7.69	−3.75	8.42	14.72	−18.64	18.96

**Bold** values indicate statistically significance (*p*<0.05) of the parameters.

CI was expressed as C_M_ for actual use of services; C_N_ for need-expected services; HI (Horizontal Inequity) for need-standardized services.

A 1.5 times gap in actual use of outpatient services between rich and poor appeared, rising from 0.089 for the least affluent to 0.136 for the most affluent. The gap in actual use of inpatient services was even larger (2.6 times), rising from 0.043 for the least affluent to 0.113 for the most affluent. This formed a sharp contrast with the higher expected need of poorer patients. The actual use of inpatient services by the respondents in the lowest income quintile accounted for only 53% of their expected need. Whereas, the actual use of inpatient services by the respondents in the highest income quintile (0.113) was 1.8 times of their expected need (0.063). Obviously, underuse of services by the poor and overuse of services by the rich coexisted (Table 2).

Large income-related inequity was evident as indicated by the distribution of need-standardized use of services. The gap in use of services between the rich and the poor, after adjustment with health need actually increased. A 1.6 times gap (rising from 0.084 for the least affluent to 0.134 for the most affluent) for use of outpatient services and 3.8 times gap (rising from 0.031 for the least affluent and 0.119 for the most affluent) for use of inpatient services was demonstrated (Table 2).

A substantial proportion of respondents with NCD forsook their otherwise needful health services, both outpatient and inpatient. Respondents with lower income were more likely to be influenced by financial difficulties. Almost 22.7% of those in the lowest quintile did not seek medical attention when needed, much higher than the 9.9% of those in the highest quintile. Similarly, 47.8% of those in the lowest quintile refused hospital admissions, much higher than the 28.1% of those in the highest quintile. The denied services were most likely to be associated with financial difficulties, which respectively accounted for 87.4% of the lowest quintile and 59.8% of the highest quintile declined or avoided inpatient services. Meanwhile, 54.5% of the least affluent and 30.6% of the most affluent attributed financial difficulties to their declined outpatient services (Table 3).

**Table 3:** Reasons for denied inpatient and outpatient services for the NCD patients across income quintiles.

	Quintile of Household living standard				
	I (Poorest)	II	III	IV	V (Richest)
% of denied use of outpatient services (over two-week)*	22.7%	18.8%	12.4%	10.9%	9.9%
**Reasons for not using outpatient services over two weeks (%)**					
Felt minor illness	30.1	34.9	49.5	43.4	47.2
Financial difficulties	54.5	39.6	28.9	30.2	30.6
Lack of time	2.8	1.9	4.1	3.6	3.7
Traffic inconvenience	0	0	0	0	0
Effective treatment not available	9.1	17.9	13.4	10.8	8.3
Other reasons	3.5	5.7	4.1	12	10.2
% of refused hospital admission*	47.8%	40.8%	40.7%	35.2%	28.1%
**Reasons for not using inpatient services over one year (%)**					
Felt not necessary	4.9	7.5	8.4	17.3	15.7
Effective treatment not available	3.8	5.3	3	4	6.1
Financial difficulties	87.4	74.3	72.9	68.6	59.8
Poor hospital services	0	0	0	0	0.3
Lack of time	2.7	2.2	10.8	4	4.1
No beds available	0	0.5	0	0.7	3.2
Other reasons	1.2	10.2	4.9	5.4	10.8
**Inpatient reimbursement rates for NCD patients enrolled in insurance schemes (%)**					
Patients Enrolled in NCMS	39.7	27.6	31.9	26.7	25.8
Patients Enrolled in MIUE	43.9	44.1	58.7	58.7	52.2
Patients enrolled in MIUR	46.7	30.5	30.9	35.4	33.9
**OOP medical expenditure as a percentage of non-food household expenses (%)**					
Patients Enrolled in NCMS	40.3	35.4	28.6	21.4	29.9
Patients Enrolled in MIUE	60.0	54.3	40.7	35.9	22.9
Patients enrolled in MIUR	42.6	58.6	30.9	37.4	31.8

*p<0.01, compared across the five income quintile groups.

Despite high enrolment in health insurance schemes, compensation for medical expenses from those schemes was generally low (Table 3). The reimbursement rate for enrollees of the rural New Cooperative Medical Scheme (NCMS) declined as household income increased, changing from 39.7% for the least affluent to 25.8% for the most affluent. The Medical Insurance for Urban Residents (MIUR) offered a higher reimbursement rate for the least affluent (46.7%) as compared to the most affluent (30.5%–35.4%). However, higher reimbursement rates (52.2%–58.7%) were found for the more affluent enrollees of the Medical Insurance for Urban Employees (MIUE) as compared with their less well off counterparts (43.9%–44.1%).

The NCD patients with low income paid a disproportionally higher amount of medical expenditure out of pocket (OOP) as a percentage of their income. The OOP medical expenditure as a percentage of non-food household expenses declined as household income increased, changing from 40.3% to 29.9% for NCMS enrollees, 60.0% to 22.9% for MIUE enrollees and 42.6% to 31.8% for MIUR enrollees, respectively (Table 3).

### 3. Factors contributing to income-related inequalities

Income-related inequalities as measured by CI in outpatient and inpatient services were decomposed into contributions of individual determinants, including both need and non-need variables (Table 4). The marginal effect (β_k_) of each variable was obtained by running regression based on Equation 2, which indicates an association between a particular variable and use of services. Those with a positive sign demonstrate a positive association with use of services, and vice versa. In addition, the larger the absolute value a β_k_ is, the more substantial the association is.

**Table 4:** Decomposition of income-related inequalities in use of outpatient and inpatient services by need and non-need variables.

Determinants	Outpatient Services		Inpatient Services	
	Marginal effects (β_k_)	Contribution%	Marginal effects (β_k_)	Contribution%
**Gender and Age**				
**Men**				
15–24	−0.041	0.4%	**−0.046**	0.3%
25–34	0.018	−0.1%	−0.007	0.0%
35–44	**0.124**	0.6%	−0.005	0.0%
45–54	**0.174**	7.8%	0.031	0.9%
55–64	**0.230**	−6.6%	**0.071**	−1.3%
65-	**0.262**	0.5%	**0.089**	0.1%
**Women**				
15–24	Reference			
25–34	0.046	−0.7%	−0.020	0.2%
35–44	0.110	0.5%	−0.015	0.0%
45–54	**0.202**	8.2%	0.014	0.4%
55–64	**0.239**	−2.3%	0.039	−0.2%
65-	**0.251**	−4.6%	0.058	−0.7%
**Health Status**				
**Limitation of daily activities**				
Yes	**0.015**	−2.4%	**0.043**	−4.4%
No	Reference			
**Self-perceived health**				
Very poor	**0.105**	−0.6%	**0.082**	−0.3%
Poor	**0.096**	−1.9%	**0.050**	−0.6%
Fair	**0.053**	−5.4%	**0.024**	−1.5%
Good	**0.018**	1.9%	−0.001	0.0%
Excellent	Reference			
**Socioeconomic Status**				
**Education**				
Illiterate	Reference			
Primary school	−0.002	0.2%	0.003	−0.2%
Secondary school	−0.011	−4.7%	−0.002	−0.5%
University	0.003	0.9%	0.000	0.0%
**Occupation**				
Student	0.007	0.0%	−0.020	0.0%
Unemployed	Reference			
Peasant	0.001	−0.8%	−0.003	1.7%
Worker	0.015	3.9%	−0.006	−0.9%
Self-employed	0.019	3.1%	**−0.015**	−1.5%
Manager/Technician/Clerk	0.011	9.1%	−0.007	−4.0%
Other	**0.034**	0.7%	0.000	0.0%
**Marital status**				
Unmarried	Reference			
Married	0.014	2.9%	**0.021**	2.7%
Divorced/widow	0.020	−3.0%	0.019	−1.7%
Other	−0.004	0.0%	0.025	0.0%
**Household income**				
Quintile I (Poorest)	Reference			
Quintile II	0.008	−7.8%	**0.021**	−11.9%
Quintile III	0.010	0.0%	**0.028**	0.1%
Quintile IV	**0.015**	14.0%	**0.050**	29.2%
Quintile V (Richest)	**0.038**	71.3%	**0.093**	108.0%
**Location of residence**				
East	**0.029**	19.5%	**−0.017**	−7.0%
Middle	0.003	−0.4%	**0.013**	−1.1%
Western	Reference			
**Healthcare Accessibility**				
**Insurance**				
MIUE	0.015	16.1%	**0.018**	12.1%
FMC	0.005	0.7%	**0.044**	3.4%
MIUR	**0.026**	0.7%	0.017	0.3%
NCMS	**0.021**	−24.3%	**0.015**	−10.7%
Other insurance	−0.013	−0.2%	**0.050**	0.6%
No insurance	Reference			
**Shortest distance to health facilities**				
≤5 km	Reference			
>5 km	**−0.027**	3.0%	−0.001	0.1%
Time to the nearest health facilities*				
≤30 min	Reference			
>30 min	−0.001	0.2%	0.001	−0.1%

**Bold** values indicate statistical significance (p<0.05) of the estimates of marginal effects.

MIUE: Medical Insurance for Urban Employees;

FMC: Free Medical Care;

MIUR: Medical Insurance for Urban Residents;

NCMS: New Cooperative Medical Insurance Scheme for Rural Residents.

Regardless of outpatient and inpatient services, the β_k_ showed that older age and poorer health status were associated with an increased use of services (Table 4). However, those need variables were not able to explain the full extent of inequality. In fact, disparities in household income and access to health insurance schemes were the greatest contributors to the inequality in health services among the NCD patients. A positive value of “% contribution” as shown in Table 4 signifies a contribution in favor of the affluent, while a negative value of “% contribution” indicates a contribution in favor of the poor. Household income made alarmingly high contributions in favor of rich, including 71.3% of pro-rich CI for outpatient services and 108.0% pro-rich CI for inpatient services from the highest quintile of household income. Disparities in access to medical insurance schemes came second in contributing to inequality in health services. Although the NCMS made a pro-poor contribution for both outpatient (−24.3%) and inpatient (−10.7%) services, the other insurance schemes all made a pro-rich contribution (ranging from 0.6% to 12.1%). In addition, residing in eastern regions made a significant (19.5%) pro-rich contribution to use of outpatient service, but a pro-poor (−7.0%) effect on use of inpatient services.

## Discussion

Significant income-related inequity in use of health services by people with NCD exists in China. Despite a greater need of the less affluent for health services (Figure 1 and Figure 2), they have been disproportionately deprived of health services due to their socioeconomic situation. This study revealed a 1.6 times gap in outpatient service utilization and a 3.8 times gap in inpatient service utilization between the lowest income quintile and the highest income quintile of people after adjustment due to health need. Only 53% of necessary inpatient services for the poorest were delivered. By contrast, the richest used 1.8 times of need-expected inpatient services. The horizontal inequity indices also indicate a more substantial income-related inequity in inpatient services (HI_i_ = 0.253) compared with outpatient services (HI_o_ = 0.089) (Figure 1 and Figure 2).

This study also proved our hypothesis that health inequality could be underestimated by mixing study subjects with and without NCD. Indeed, the horizontal inequity indices of the people with NCD (HI_o_ = 0.089, HI_i_ = 0.253) are higher than the average of general public (HI_o_ = 0.017, HI_i_ = 0.207).

The levels of horizontal inequity in our study population are higher than those of Thai (HI_o_ = −0.067, HI_i_ = −0.061) [45] and Australia (HI_o_ = 0.019, HI_i_ = −0.083) [46]. More importantly, the Chinese health system still favours the affluent, whereas in Thailand and Australia they have become in favour of the less affluent due to the universal coverage of health care.

The decomposition analysis showed that financial factors have the most significant association with inequality in health services among the people with NCD in China. The pro-rich contributions made by household income were alarmingly high: 71.3% CI for outpatient services and 108.0% CI for inpatient services came from the highest quintile of household income. By 2008, about 90% of Chinese residents had been covered by health insurance [4], [19]. How can financial difficulties still be associated with such significant inequity in health services? First, despite rapid accumulation of social wealth, income gap between the rich and the poor is widening in China at an alarming speed. The income Gini coefficient reached 0.48 in 2008 [47], exceeding the internationally recognized warning level. The most affluent 10% of Chinese families collective wealth was equivalent to 65 times of those at the least affluent 10% in 2008 [48]. Second, despite decades of almost miraculous economic growth due to market-oriented reforms, unfortunately, the Chinese government has failed to ensure equal attention to wealth re-distribution and social insurance and safety net. Without those social protection measures, individuals and families have become a major bearer of financial risks resulted from catastrophic events such as serious diseases. This is not uncommon in a market dominated system. In the USA, for example, 47.6% of inequality in outpatient services is a result from income disparities [49]. Third, the Chinese system has actually resulted in an enlarged gap in ability to pay for health services between the rich and the poor. This study found that the richer people tended to overuse health services, especially the more expensive inpatient services. On the other hand, poorer people tended to underuse health services. Our data demonstrated that 22.7% of the people in the poorest income quintile did not seek medical attention when needed, 54% of which were caused by financial difficulties. Meanwhile, as high as 47.8% of those in the poorest income quintile denied hospital admissions, 87.4% of which were caused by financial difficulties. Finally, insufficient financial investment from both national and provincial governments and fee-for-service payment mechanisms for health providers (and perhaps also the high demand from the affluent) have altered the market equilibrium for medical cost [50], which may in turn compounds the financial difficulties of the poor. In the last three decades, governmental subsidies to public hospitals have been reduced to less than 8% of total revenue of public hospitals [51]. Public hospitals have to rely on user charges for cost recovery [52]. These fee-for-service arrangements encouraged health workers to provide more (but often unnecessary) services [13]. The supplier-induced demand has fuelled the rise of health expenditure and increased the financial burden of consumers. The NCD patients may become the first to feel the burden, because medical expenditure incurred on the NCD patients were 2.9 and 1.5 times of the average for outpatient and inpatient services, respectively.

It has been widely accepted that health insurance can help reduce inequality in use of health services [17], [19], [20]. Our study revealed that health insurance was the second largest determinant of inequality in health services; however, some medical insurance schemes such as the MIUE actually made a pro-rich contribution to the inequality in health services (16.1% for outpatient and 12.1% for inpatient). There are several possible reasons for this. The compensation rate for MIUE enrollees fell short of government expectation (75%) [53]. Our study showed that consumers had to pay about half of medical expenses out of pocket (OOP). The average inpatient medical expenditure for the people with NCD had reached 1672 US$ in 2008 and consumers shared 60% of the charge. The OOP medical payments amounted to 60% of non-food expenses of the household in the poorest quintile, much higher than that of the household in the richest quintile (22.9%). In addition, richer people enjoyed a higher reimbursement rate for inpatient services (52.2%–58.7%) than their poorer counterparts (43.9%–44.1%). These were associated with underuse of inpatient services by the poor and overuse of inpatient services by the rich. Our results showed that the richer people used more inpatient services than what they needed. The actual use of inpatient services by the richest quintile was 0.113, 1.8 times the need-expected (0.063). The overuse of inpatient service by the rich is perhaps also stimulated by the strong focus of the MIUE on hospital services, offering the highest reimbursement rate for inpatient services among all insurance schemes.

Unlike the MIUE, the NCMS made a contribution in favor of the poor (−24.3% for outpatient services and −10.7% for inpatient services). This finding is consistent with studies undertaken by others [54]. The NCMS covered a very large rural population (91.5% of rural residents), with 585 million having their medical bills reimbursed in 2008 [51]. The widespread financial benefits were achieved through a primary care-oriented approach. The NCMS offered higher levels of reimbursement for services in township facilities (40%–50%) than in county hospitals (15%–45%) [23], [55], which encouraged people to seek primary care services closer to their communities. Our data showed that 41.3% of NCMS enrollees chose township facilities for inpatient services for their NCD, resulting in much cheaper bills (The average cost for township facilities was 174.8 US$). The average expenditure for inpatient services was 654 US$ for NCMS enrollees, 1018 US$ less than the average for MIUE enrollees. In addition, the reimbursement policy of NCMS was also in favor of the poor (25.8% for the richest, 39.7% for the poorest). Nevertheless, the overall level of reimbursement of the NCMS was very low (30.8%, lowest in the insurance schemes), which had restricted its role in improving equality in health services.

Serious flaws exist in the design of China's health insurance schemes. Both MIUE and NCMS comprise two components: individual medical saving accounts (IMSA) and social pooling account (SPA). The SPA are intended to cover inpatient costs while the IMSA are to cover outpatient costs and to pay for deductibles in inpatient fee. These multi-tier insurance schemes are inequitable in themselves. In 2008, the per capita fund in the IMSA reached 160 US$ for MIUE enrollees, much higher than the 1.2 US$ for NCMS enrollees [13]. Since NCD patients need life-long medical treatment, the IMSA can only offer very limited assistance because funds in the IMSA are not intended to be shared amongst enrollees. Given this, many NCD patients, especially those from a household with higher income, turned to inpatient services so that they could at least get some compensation from the SPA. But they still faced greater financial hurdles imposed by the OOP payment requirement (deductibles, co-payment requirement and maximum amount of money that the insurance fund would pay for). As a result, the poor have to seek cheaper services and may eventually give up their needed services. Studies have found that rich people are more likely to visit expensive tertiary health facilities than the poor [24], [56], although many of the services are deemed unnecessary and necessary services could be provided in primary care facilities. Unfortunately, services offered in primary care facilities are often deemed to be inappropriate or of poor quality [57], which in turn encourages more people (especially those who can afford) to seek perhaps unnecessary services from tertiary facilities. OOP spending has been believed to be the biggest barrier for reducing urban-rural health service gap [17].

## Limitations

There are a few limitations in this study. First, the NHHS is carried out every five years in China. This study has not been able to analyze the most updated progress in recently years of health reform in China. Second, two binary indicators were used for measuring use of outpatient and inpatient services. This can only capture the extensive margin of health service utilization. Further studies are needed to investigate the intensive margin of health service utilization, using indicators in relation to financial burden and quality of services. Third, the NHHS is a cross-sectional survey, which prevents us from making any causal conclusions in the analyses. Some independent variables may be endogenously determined: for example, sicker people who utilize more healthcare services are more likely to be covered by health insurance, and therefore it would be hard to distinguish which factor affects the other. However, Chinese people have very limited choice of health insurance policies. Over 90% of rural residents have been covered by the NCMS. Urban insurance schemes also have strict eligibility criteria and very high coverage in their respective populations. Although empirical evidence shows that household income and insurance policies - the two main factors concerned in this study are likely to affect service use rather than the other way around, we can not exclude the possibility of endogeneity. A potential correlation in both directions of those variables will bias the coefficient estimations and lend weak support to the model for service use inequality measures and decomposition. Finally, the two week short recall period for outpatient service use may result in large measurement errors.

### Conclusion

There is strong pro-rich inequality in health services for the Chinese people with NCD. Disproportionately high use of health services by the most affluent coexists with underuse of health services by the least affluent. The income-related horizontal inequity in inpatient services is much higher than that of outpatient services. Disparities in financial capacity and medical insurance entitlement are the two main factors contributing to the pro-rich inequity.

The unusual large share of contribution from financial factors to inequality in health services reflects the widening wealth gap in China and its powerful association with health services. Health policy alone is far from enough to address the inequality issue. A comprehensive social policy that encompasses a more progressive taxation package and redistribution of social wealth as well as pro-poor welfare is needed. This should include a more equitable health insurance program, which extends the pooling of resources to cover all forms of healthcare.

The current financing levels of health insurance schemes are inadequate. The general low insurance benefit level in combination with multi-tier insurance arrangements may actually exacerbate pro-rich inequity in use of health services. The design of the insurance schemes is unable to cater to the real needs of NCD patients, and may encourage NCD patients to use more expensive inpatient services instead of cheaper and more cost effective preventative and primary care.

It is important to develop a mechanism that ensures services are delivered according to the need of consumers, not their ability to pay. There is a need for the government to redirect resources to subsidize the less affluent in the insurance schemes, to increase the percentage of insurance compensation, to encourage primary care through social pooling mechanism for outpatient services, and to eliminate perverse financial incentives for health providers by changing funding instruments. There is a long way to go to achieve the government defined goal of increasing insurance compensation from 52.5% to 75% for MIUE and from 30.8% to 60% for NCMS [53].

## Supporting Information

Table S1 **Description of independent and control variables.**
(DOCX)
