# Supplementary material for: Identifying Determinants of Socioeconomic Inequality in Health Service Utilization among Patients with Chronic Non-Communicable Diseases in China
Source: PLoS One. 2014 Jun 24;9(6):e100231. doi: 10.1371/journal.pone.0100231 (PMC4069022; doi:10.1371/journal.pone.0100231)
Supplement: Table S1 — Description of independent and control variables. (DOCX) [file pone.0100231.s001.docx]

Caption：**Description of independent and control variables**

**Table S1. Description of independent and control variables**

| **Variable** | **Category** | **Coding** |
| --- | --- | --- |
| **Gender and Age (years)** |  |  |
| Men |  |  |
|  | 15-24 | 1= male (15-24), 0=otherwise |
|  | 25-34 | 1= male (25-34), 0=otherwise |
|  | 35-44 | 1 = male (35-44), 0=otherwise |
|  | 45-54 | 1 = male (45-54), 0=otherwise |
|  | 55-64 | 1 = male (55- 64), 0=otherwise |
|  | 65- | 1 = male (65- ), 0=otherwise |
| Women |  |  |
|  | 15-24 | 1 = female (15-24), 0=otherwise |
|  | 25-34 | 1 = female (25-34), 0=otherwise |
|  | 35-44 | 1 = female (35-44), 0=otherwise |
|  | 45-54 | 1 = female (45- 54), 0=otherwise |
|  | 55-64 | 1 = female (55- 64), 0=otherwise |
|  | 65- | 1 = female (65- ), 0=otherwise |
| **limitation of daily activities in last 12 months** |  |  |
|  | Yes | 1 = yes, 0=otherwise |
|  | No | 1 = no, 0=otherwise |
| **Self-rated general health** |  |  |
|  | Very poor | 1 = very poor, 0=otherwise |
|  | Poor | 1 = poor, 0=otherwise |
|  | Fair | 1 = fair, 0=otherwise |
|  | Good | 1 = good, 0=otherwise |
|  | Excellent | 1 = very good, 0=otherwise |
| **Educational attainment** |  |  |
|  | Illiterate | 1 = illiterate, 0=otherwise |
|  | Primary school | 1 = primary school, 0=otherwise |
|  | Secondary school | 1 = secondary school, 0=otherwise |
|  | University | 1 = university, 0=otherwise |
| **Occupation** |  |  |
|  | Student | 1 = student, 0=otherwise |
|  | Unemployed | 1 = unemployed, 0=otherwise |
|  | Peasant | 1 = peasant, 0=otherwise |
|  | Worker | 1 = worker, 0=otherwise |
|  | Self-employed | 1 = self-employed, 0=otherwise |
|  | Manager/Professional/Clerk | 1 = manager/professional/clerk, 0=otherwise |
|  | Other | 1 = others, 0=otherwise |
| **Marital status** |  |  |
|  | Unmarried | 1 = unmarried, 0=otherwise |
|  | Married | 1 = married, 0=otherwise |
|  | Divorced/widowed | 1 = divorced or widowed, 0=otherwise |
|  | Other | 1 = other, 0=otherwise |
| **Household income** |  |  |
|  | Quintile I (poorest) | 1 = quintile I, 0=otherwise |
|  | Quintile II | 1 = quintile II, 0=otherwise |
|  | Quintile III | 1 = quintile III, 0=otherwise |
|  | Quintile IV | 1 = quintile IV, 0=otherwise |
|  | Quintile V (richest) | 1 = quintile V, 0=otherwise |
| **Location of residence** |  |  |
|  | Eastern region | 1 = eastern region , 0=otherwise |
|  | Middle region | 1 = middle region, 0=otherwise |
|  | Western region | 1 = western region, 0=otherwise |
| **Health insurance policies** |  |  |
|  | MIUE | 1 = MIUE, 0=otherwise |
|  | FMC | 1 = FMC, 0=otherwise |
|  | MIUR | 1 = MIUR, 0=otherwise |
|  | NCMS | 1 = NCMS, 0=otherwise |
|  | Other insurance | 1 = other health insurances, 0=otherwise |
|  | No insurance | 1 = no health insurance, 0=otherwise |
| **Distance to the nearest health facilities** |  |  |
|  | ≤5km | 1 = distance ≤5km 5km, 0=otherwise |
|  | >5km | 1 = distance >5km, 0=otherwise |
| **Time to the nearest health facilities** |  |  |
|  | ≤30min | 1 = time ≤ 30 minutes, 0=otherwise |
|  | >30min | 1 = time >30 minutes, 0=otherwise |

MIUE, Medical Insurance for Urban Employees;

FMC, Free Medical Care

MIUR, Medical Insurance for Urban Residents

NCMS, New Cooperative Medical Insurance Scheme for Rural Residents
